# Negative emotionality downregulation affects moral choice but not moral judgement of harm: a pharmacological study

**DOI:** 10.1038/s41598-024-51345-8

**Published:** 2024-01-12

**Authors:** Roger Marcelo Martinez, Shih-Han Chou, Yang-Teng Fan, Yu-Chun Chen, Kah Kheng Goh, Chenyi Chen

**Affiliations:** 1 Graduate Institute of Injury Prevention and Control, College of Public Health, Taipei Medical University, 250 Wu-Hsing Street, Taipei, 110 Taiwan; 2 School of Psychological Sciences, National Autonomous University of Honduras, Tegucigalpa, Honduras; 3 Division of Neurosurgery, Department of Surgery, Wan Fang Hospital, Taipei Medical University, Taipei, Taiwan; 4 Department of Physical Medicine and Rehabilitation, Wan Fang Hospital, Taipei Medical University, Taipei, Taiwan; 5 Department of Physical Medicine and Rehabilitation, Taipei Medical University Hospital, Taipei Medical University, Taipei, Taiwan; 6 Graduate Institute of Medicine, Yuan Ze University, Taoyuan, Taiwan; 7 Department of Physical Education, National Taiwan University of Sport, Taichung, Taiwan; 8 Department of Psychiatry, Wan-Fang Hospital, Taipei Medical University, Taipei, Taiwan; 9 Department of Psychiatry, School of Medicine, College of Medicine, Taipei Medical University, Taipei, Taiwan; 10 Psychiatric Research Center, Wan Fang Hospital, Taipei Medical University, Taipei, Taiwan; 11 The Innovative and Translational Research Center for Brain Consciousness, Taipei Medical University, Taipei, Taiwan; 12 Neuroscience Research Center, Taipei Medical University Hospital, Taipei, Taiwan; 13 Graduate Institute of Mind, Brain and Consciousness, College of Humanities and Social Sciences, Taipei, Taiwan

**Keywords:** Neuroscience, Cognitive neuroscience, Emotion, Social neuroscience

## Abstract

Previous neuroscientific research has expounded on the fundamental role played by emotion during moral decision-making. Negative emotionality has been observed to exert a general inhibitory effect towards harmful behaviors against others. Nevertheless, the downregulation of negative affects at different levels of moral processing (e.g. impersonal versus personal moral dilemmas) alongside its possible interactions with other factors (e.g. perspective taking) hasn’t been directly assessed; both of which can assist in predicting future moral decision-making. In the present research, we empirically test (Study 1, N = 41) whether downregulating negative emotionality through pharmacological interventions using lorazepam (a GABA receptor agonist), modulate the permissibility of harm to others –i.e. if participants find it more morally permissible to harm others when harm is unavoidable (inevitable harm moral dilemmas), than when it may be avoided (evitable harm moral dilemmas). Furthermore, using another sample (Study 2, N = 31), we assess whether lorazepam’s effect is modulated by different perspective-taking conditions during a moral dilemma task –e.g. “is it morally permissible for *you* to […]?” (1st person perspective), relative to “is it morally permissible for [x individual] to […]?” (3rd person perspective)–, where the outcome of the different scenarios is controlled. The results of both studies converge, revealing an emotion-dependent, rather than an outcome-dependent, pharmacological modulation. Lorazepam only influenced interpersonal moral judgments when not modulated by the evitable/inevitable condition. Furthermore, there was a significant interaction between perspective-taking and drug administration, as lorazepam exerted a larger effect in modulating moral choices rather than moral judgements.

## Introduction

Owing it almost entirely to classical philosophy, morality and moral decision-making have been traditionally presumed to be solely the fruits of rational thought and deliberation^1^. Nevertheless, recent research has observed that emotion and negative emotionality^2^, both of which can be rooted in interoception and the bodily sense of self^3^, play fundamental roles in moral reasoning.

Regarding negative emotionality, studies have shown that it is able to prompt reluctance towards engaging in anti-social behaviors^4^. Individuals who tend to act and adhere to societal norms and social pressure, are more prone to have increased levels of negative affect as a function of their aversion towards risk and uncertainty^4–7^. Furthermore, the “gut” feeling commonly described when conflicting with others or incurred in by the expectation of harming another person^8^, has been observed to be a common trigger for negative emotionality, as well as anxiety^9,10^. What’s more, research using the moral dilemma task, has also observed that psychopaths possessing low-anxiety are more likely than either high-anxiety psychopaths and non-psychopathic participants to endorse directly harmful behaviors in moral-personal dilemmas. Both clinical observations and criterion group studies of psychopaths therefore suggest that anxiety may play a key role in modulating preferences for direct harmful acts in moral-personal dilemmas. Nonetheless, the latter conclusions must be taken with caution, as they have been determined by using correlational data, which cannot test for a causal role of anxiety in moral decision-making^11^.

Interestingly, studies using false feedback paradigms –where subjects listened to a fake-fast or a fake-normal heartbeat posing to be that of themselves– have shown that an increased heart rate (even when fake) predisposed participants to volunteer more towards charitable causes, and curbed down lying for selfish purposes^12^. Moreover, research has also observed that both physical and moral disgust equally provoke facial expressions of disgust^13,14^, owing to the fact that brain regions that deal with physical and moral disgust overlap with one another^15,16^. But how is it that morality and moral decision-making, both of which have been intimately linked to neural and cognitive processes (being them through rationalist, intuitionist, dual or dynamic models)^17,18^, interact with interoception and biological regulation –i.e. bodily functions? One prominent hypothesis explaining such findings posits that emotional experiences guide morality and moral decision-making by making use of bodily signals or ‘somatic states’, which exert influence over conscious responses and decisions. Such hypothesis has come to be known as the somatic marker hypothesis^19,20^. In line with this hypothesis, studies using the noradrenergic beta-adrenoceptor antagonist propranolol –originally developed to treat heart and circulatory-related conditions^21,22^– have observed the drug’s ability to influence moral decision-making; as its administration reduced participants’ utilitarian responses in a moral dilemma task. This is due to propranolol being able to suppress noradrenergic receptors, reduce heart rate, and lower overall emotional arousal, which leads to an increase in aversion towards harming others^20,21^, as well as a decrease in aggression^20,23^. Furthermore, research using the selective serotonin reuptake inhibitor (SSRI) citalopram, showed that increasing serotonin via SSRI administration promotes prosocial behaviors by amplifying harm aversion^24^, owing to the fact that serotonin seems to be essential for translating aversive stimuli and distress cues into behavioral inhibition and withdrawal responses^25,26^. Moreover, envisioning harmful behaviors directed towards others engages neural areas such as the ventromedial prefrontal cortex, anterior cingulate cortex, the striatum and the amygdala, all of which possess dense serotonergic projections^27^. Conversely, it has been observed that pharmacological enhancements of dopamine function increase harm aversion towards oneself, but decrease harm aversion towards others; thus, reducing altruism and prosocial behaviors^28^.

The anxiolytic drug lorazepam is a high-potency 3-hydroxy benzodiazepine prescribed for the relief of anxious symptomatology^29^, as it enhances GABA release in the brain by binding to the GABA receptors. Past research has also demonstrated that there is a dose-dependent decrease in insula and amygdala activation during emotional processing following lorazepam administration^30^. This is in line with the current neuroscientific consensus which posits the amygdala as a key neural region implicated in fear conditioning, and the insula as a key brain region involved in the modulation of affective and aversive interoceptive processing^30,31^. Furthermore, insular interoceptive processing has been observed to be correlated with GABA concentrations in this same brain region, to such degree that both GABA and interoceptive signal changes in the insula predict the intensity of depressed affect in individuals^32^. Interestingly, when it comes to morality, psychopharmacological research in human subjects has demonstrated that lorazepam incurs in a dose-dependent increase in the participants' willingness to endorse responses that directly harm others in moral-personal dilemmas, regardless of whether the motivation for those harmful acts is deontological or utilitarian, this due to the drug’s ability to reduce threat intensity during the moral dilemma task^11^. Therefore, it is strongly suggested that anxiolytic drugs cause their effects by altering GABAergic modulation and activity in neural regions involved in emotional negativity and anxious symptomatology, as well as, presumably, interoceptive processing^31,33^.

The general objective of the present double-blind, crossover design, placebo-controlled study is that of evaluating lorazepam’s GABAergic modulation on moral decision-making. In order to reach this objective, this study aims to tackle the limitations of previous studies delving into the GABAergic modulation of neural regions involved in moral reasoning and moral decision-making. More specifically, Perkins, et al.^11^’s study, which was able to observe that lorazepam administration prompted participants to endorse direct harmful acts more readily when engaging in moral-personal dilemmas; consequently, arriving at the conclusion that lorazepam’s GABAergic modulation within emotional centers in the brain decreases inhibitions when partaking in the moral dilemma task, thus, effectively increasing the participants’ ruthlessness. Nevertheless, said study would have highly benefitted from introducing perspective taking (1st vs. 3rd person perspective) among the variables in its experimental design, in order to observe lorazepam’s interaction on the moral dilemma task depending on personal perspective-taking. In the same manner, we think that said study might also benefit from analizing the differential effects of lorazepam in evitable and inevitable harm in the moral dilemma task, since, and despite their shared theoretical relevance to emotional processes, no study has examined said associations between inevitability of bringing about harm and fear conditioning, as well as aversive interoceptive processes. As such, we have included these new variables in the experimental design of the present study, as to complement Perkins et al.’s findings. We hypothesize, as Perkins et al. did before us, that lorazepam will be able to modulate the participants’ willingness to commit (or not) certain kinds of moral acts. Specifically, our primary hypothesis (H1) is that our study will be able to replicate Perkins et al.’s results concerning the interaction between lorazepam and the degree of physical involvement of the participant in the moral dilemma task –e.g. non-moral dilemma vs. personal dilemma vs. impersonal dilemma–, with lorazepam exerting a larger effect on personal dilemmas versus non-moral and impersonal dilemmas (study 1). For our second hypothesis (H2), we anticipate a larger lorazepam effect for the outcome of evitable harm. Personal moral dilemmas which include evitable harm tend to elicit a greater and more pronounced conflict between deontological and utilitarian styles of moral decision-making (Study 1). Third, we hypothesize (H3) that the effects of lorazepam will interact with choice-of-action endorsement and moral judgment during the moral dilemma task evaluation, with choice-of-action endorsement being operationalized as the emotional processing of moral dilemmas dependent of 1st person perspective-taking (e.g. “is it morally permissible for *you* to press the switch?”), and moral judgement being operationalized as the emotional processing of moral dilemmas dependent of 3rd person perspective-taking (e.g. “is it morally permissible for [X individual] to press the switch?”) (study 2). Specifically, we anticipate a larger lorazepam effect for choice-of-action endorsements than for moral judgements.

## Study 1

### Methods

#### Participants

Forty-one healthy volunteers (23 males), aged between 21 and 31 (mean ± SD: 23.63 ± 2.44) years, were recruited from the community via online posts and paper flyers. All participants were Han Chinese and right-handed.

Participants were screened for major psychiatric illnesses (e.g. general anxiety disorder) by the Structured Clinical Interview for DSM-IV Axis I Disorders (SCID-I) and excluded if there was evidence of comorbid neurological disorders (e.g. dementia, seizures), history of head injury, and alcohol or substance abuse or dependence within the past 5 years. The sample size was estimated using G*Power^34^ prior to the data collection. To detect a medium effect size^11^ for main effects in the ANOVA with 95% power (f = 0.3, with alpha = 0.05, number of groups = 2, number of measurements = 3), a sample size of 32 participants was required. All participants had normal vision or corrected normal vision. They participated in the study after providing written informed consent. This study was approved by the Ethics Committee of the National Yang-Ming Chiao-Tung University (YM104041E), and conducted in accordance with the Declaration of Helsinki. This study was not preregistered.

#### Procedure

**Figure 1:**
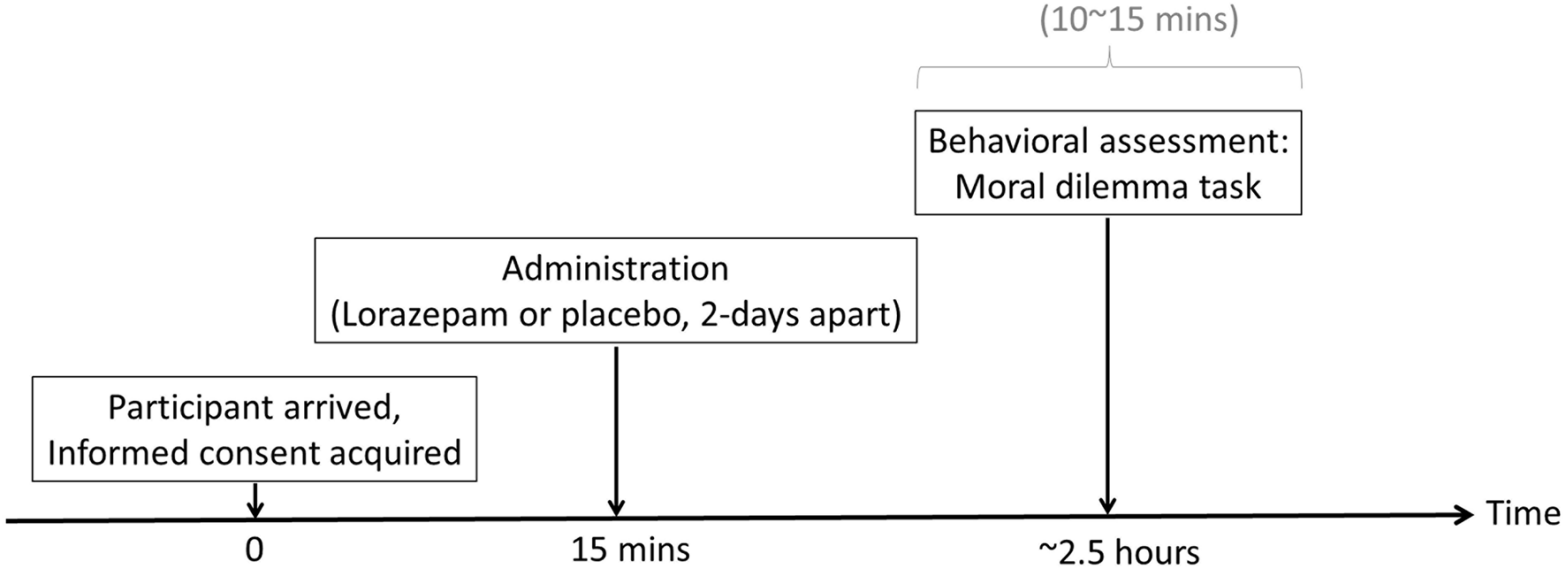
Diagrammatic representation of the study design and data collection process.

In this double-blind, placebo-controlled, crossover design study, participants received a single 0.5-mg dose of lorazepam (ATIVAN) on one day, and a single dose of placebo (i.e. vitamin E) on another day. A crossover study is a longitudinal study in which subjects receive a sequence of different treatments. Every participant received 0.5 mg lorazepam one day and placebo on another day. The two sessions were scheduled at least 2 days apart. Both lorazepam and placebo were administered orally. The experimental sequence of lorazepam and placebo administration was counter-balanced between participants through a Latin square design, which randomizes through having equal number of AB (lorazepam-placebo) and BA (placebo-lorazepam) sequences. Thus, half of the participants went first through the lorazepam session, and half of them went first through the placebo session. To coincide with the pharmacokinetics of lorazepam^35^, participants filled out the moral dilemma to access the moral permissibility of harm approximately 2-h after treatment administration (Fig. 1).

### Materials

#### Moral dilemma task

Based on previous work^8,36–38^ [https://www.cell.com/cms/10.1016/j.neuron.2004.09.027/attachment/86c8c813-bafc-49fe-8393-be1f9226358d/mmc1.pdf], forty-eight moral dilemmas were selected in order to make two versions (Version A and Version B) of the moral judgment task balanced on emotional intensity^8,39^. Each version consists of twenty-four dilemmas, which include nine non-moral dilemmas and fifteen moral dilemmas – these fifteen subdivide into five impersonal dilemmas, and ten personal dilemmas. Furthermore, each of the fifteen moral dilemmas consist of a predicament were the responder needs to decide on whether they would induce (being it directly or indirectly) harm or death to another person in order to save a larger number of people. Impersonal dilemmas involve indirect harm (e.g. flipping a switch), whereas personal dilemmas include harm through direct physical contact (pushing a stranger), such as in the Trolley dilemma. Personal dilemmas are further divided into dilemmas in which the death or harm to the victim is inevitable or evitable. Moral permissibility judgments are higher for transgressions that lead to inevitable harm, due to the principle of lesser evil^40,41^. In each dilemma, participants were asked to definitively choose between 'yes' and 'no' in response to the moral permissibility of the behaviors described in the scenario. The sequence of dilemma version used for lorazepam and placebo sessions was counter-balanced between participants through a Latin square design, which randomizes through having equal number of AB (Version A-Version B) and BA (Version B-Version A) sequences in both lorazepam and placebo sessions. Since one of the objectives of Study 1 was to replicate the findings of Perkins et al.^11^, the moral dilemmas presented to participants were framed in the first-person perspective, consistent with the approach used in Perkins et al.'s research, without explicitly priming this perspective.

The dilemmas were translated from English to Chinese, and then translated back from Chinese to English and checked for consistency by a native English speaker. Participants read the moral dilemmas in a paper booklet provided to them by the experimenters, and responded to the dilemmas with a decision of yes (endorsement of action) or no (disapproval of the action) in a separate answer sheet. All participants completed the moral dilemma task at their own pace. Moral permissibility is assessed by the percentage of harm endorsements in each dilemma type, calculated by dividing the number of trials with harm endorsements by the total number of trials. The endorsement rate (%) for each dilemma type is then used as the dependent variable for further analyses.

### Results

Table 1 presents descriptive statistics for the outcomes of endorsement rate by dilemma type and drug condition for the whole sample (n = 41). Given that the endorsement rates across participants are not normally distributed, we utilized non-parametric analysis for related samples in a within-subject design. This involved applying the Wilcoxon signed ranks test for two-sample comparisons and the Friedman Test for comparisons involving more than two groups (please see supplementary results, Tables S1 and S2 for the results of sensitivity tests using parametric analysis of repeated ANOVA).

**Table 1:** Descriptive statistics for moral permissibility [Study 1, N = 41 (23 males), between 21 and 31 (mean ± SD: 23.63 ± 2.44) years of age] by dilemma type and drug condition.

Moral permissibility	Placebo	Lorazepam	Z value	P value
Measurements (%)	Mean ± SE	Mean ± SE		
Nonmoral	63.69 ± 5.47	55.28 ± 5.37	0.293	0.769
Moral-impersonal	63.9 ± 4.95	48.29 ± 4.47	1.802	0.069
Moral-personal (all)	33.59 ± 2.51	40.33 ± 2.32	2.705	0.007
Moral-personal-evitable	22.59 ± 2.23	28.17 ± 1.94	2.018	0.044
Moral-personal-inevitable	48.9 ± 4.83	58.8 ± 3.84	2.092	0.036

Moral permissibility is assessed by the percentage of harm endorsements in each dilemma type, calculated by dividing the number of trials with harm endorsements by the total number of trials. The Z value represents the results from non-parametric analyses conducted using the Wilcoxon Signed Ranks Test. This test compares two related samples in a within-subject design, specifically evaluating moral permissibility in placebo and lorazepam conditions.

#### H1: Associations between source of bringing about harm and the effect of lorazepam

An interaction between the source of bringing about harm (personal vs. impersonal) and the lorazepam effect was found. Lorazepam administration increased the endorsement of harming for the personal dilemmas (Lorazepam: 40.33 ± 2.32, Placebo: 33.59 ± 2.51, Z = 2.705, *P* = 0.007) but not for impersonal dilemmas (Lorazepam: 48.29 ± 4.47, Placebo: 63.9 ± 4.95, Z = 1.802, *P* = 0.069). This study replicated these findings from previous literature^11^. Hypotheses 1 was supported (Table 1).

#### H2: Associations between inevitability of bringing about harm and the effect of lorazepam

**Figure 2:**
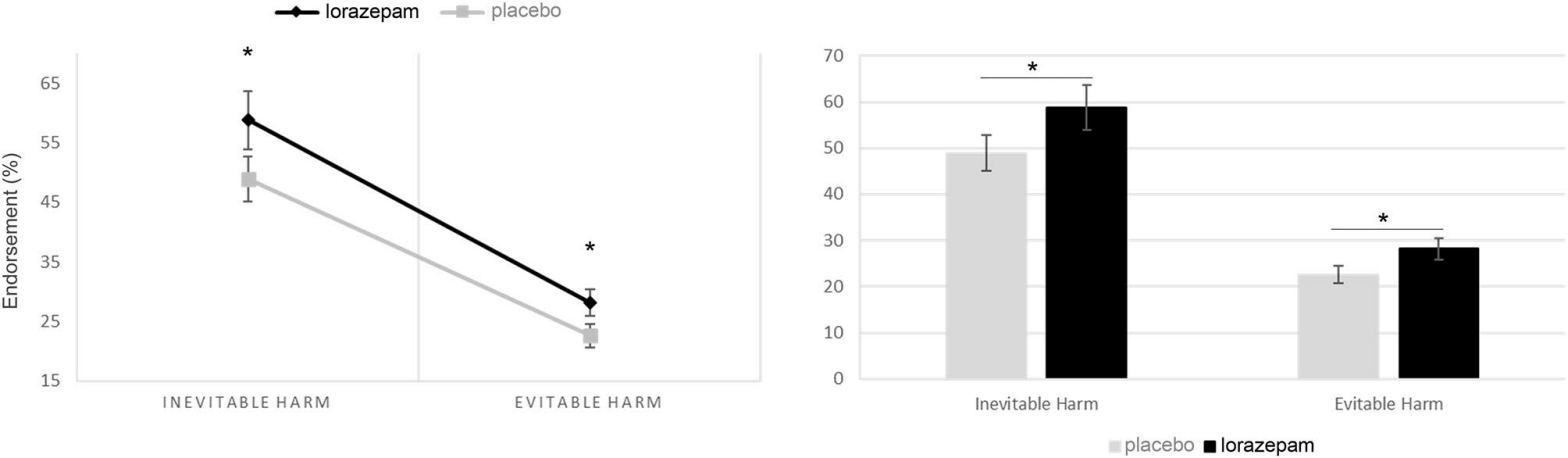
Dilemma judgements (i.e. % utilitarian responses) by treatment administration for the evitable harm and inevitable harm (*F*_1, 40_ = 0.6, *P* = 0.444).

In order to further examine whether the outcomes regarding harm in personal dilemmas interact with lorazepam administration, we compared the rates of harm endorsement under placebo and lorazepam conditions, specifically focusing on 'personal evitable harm' and 'personal inevitable harm' scenarios. Lorazepam administration had stronger endorsement for personal harming in both inevitable harm (Lorazepam: 58.8 ± 3.84, Placebo: 48.9 ± 4.831, Z = 2.092, *P* = 0.036) and evitable harm (Lorazepam: 28.17 ± 1.94, Placebo: 22.59 ± 2.23, Z = 2.018, *P* = 0.044), with similar Z value. The effect size of lorazepam administration did not change across the inevitability of bringing about harm (Table 1, Fig. 2). Hypotheses 2 was not supported.

## Study 2

### Methods

#### Participants

An independent sample of participants (N = 31, 15 males), aged between 21 and 28 (23.26 ± 1.65) years, participated in study 2 after providing written informed consent. All participants had normal vision or corrected for normal vision, were Han Chinese, right-handed, screened for major psychiatric illnesses (e.g. general anxiety disorder) by the Structured Clinical Interview for DSM-IV Axis I Disorders (SCID-I), and excluded if there was evidence of comorbid neurological disorders (e.g. dementia, seizures), history of head injury, and alcohol or substance abuse or dependence within the past five years. This study was approved by the Ethics Committee of the National Yang-Ming Chiao-Tung University (YM104041E), and conducted in accordance with the Declaration of Helsinki. This study was not preregistered.

#### Procedure

In study 2, the same double-blind, placebo-controlled, crossover design was applied. Participants received a single 0.5-mg dose of lorazepam (ATIVAN) on one day, and a single dose of placebo (i.e. vitamin E) on another day. As in study 1, The experimental sequence of lorazepam and placebo administration was counter-balanced between participants through a Latin square design, which randomizes through having equal number of AB (lorazepam-placebo) and BA (placebo-lorazepam) sequences. After treatment administration and a 2-h interval to coincide with the pharmacokinetics of lorazepam, participants filled out the moral dilemma task in order to assess their moral permissibility.

### Materials

#### Moral dilemma task

In study 2, we employed the same moral dilemma task as in study 1: two versions of the moral dilemma task (Version A and Version B), balanced in terms of emotional intensity^39^, were used to counterbalance the administration of lorazepam and placebo. Each version included five impersonal dilemmas, five personal-inevitable dilemmas, and five personal-evitable dilemmas. Notwithstanding, for study 2, we included two variations in the moral dilemma task. First, the moral dilemmas were re-written and reframed from a neutral perspective (contrary to study 1, where the moral dilemmas retained the first person perspective from Perkins et al.’s research). Second, following each moral dilemma scenario, and in order to foster third-person versus first-person perspective-taking, participants were asked two questions: 'Is it morally permissible for other people to perform the behaviors depicted in this dilemma?' (3rd person-perspective condition), and 'is ti morally permissible for you perform the behaviors depicted in this dilemma?' (1st person-perspective condition). The order of third-person and first-person perspective conditions was counterbalanced among participants using a Latin square design. This ensured an equal number of third-person-first-person and first-person-third-person sequences in both the lorazepam and placebo sessions. All participants completed the moral dilemma task at their own pace.

### Results

Table 2 presents descriptive statistics for the outcomes of endorsement of harm rate by dilemma type, drug condition, and moral perspective-taking (n = 31). Given that the endorsement rates across participants are not normally distributed, we utilized non-parametric analysis for related samples in a within-subject design. This involved applying the Wilcoxon Signed Ranks Test for two-sample comparisons and the Friedman Test for comparisons involving more than two groups (please see supplementary results, Tables S1 and S2 for the results of sensitivity tests using parametric analysis of repeated ANOVA).

**Table 2:** Descriptive statistics for moral judgement and choice [Study 2, N = 31 (15 males), between 21 and 28 (mean ± SD:23.26 ± 1.65) years of age] by dilemma type and drug condition.

Moral permissibility	Placebo	Lorazepam	Z value	P value
Measurements (%)	Mean ± SE	Mean ± SE		
Moral judgement (3rd person-perspective)				
Moral judgement (all)	51.02 ± 3.25	52.47 ± 3.45	0.47	0.638
Moral-impersonal	55.48 ± 6.06	63.87 ± 6.04	0.788	0.431
Moral-personal-evitable	33.87 ± 3.41	30.65 ± 3.2	1.388	0.165
Moral-personal-inevitable	63.71 ± 5.54	62.9 ± 5.54	0.047	0.963
Moral choice (1st person-perspective)				
Moral choice (all)	39.86 ± 2.71	48.08 ± 3.05	2.156	0.031
Moral-impersonal	44.03 ± 5.73	60.65 ± 6.12	1.48	0.139
Moral-personal-evitable	23.12 ± 2.96	27.96 ± 2.83	1.442	0.149
Moral-personal-inevitable	52.42 ± 5.72	55.65 ± 5.65	0.587	0.557

Moral permissibility is assessed by the percentage of harm endorsements in each dilemma type, calculated by dividing the number of trials with harm endorsements by the total number of trials. The Z value represents the results from non-parametric analyses conducted using the Wilcoxon signed ranks test. This test compares two related samples in a within-subject design, specifically evaluating moral permissibility in placebo and lorazepam conditions.

#### H3: Associations between moral perspective-taking and the effect of lorazepam for moral dilemmas

**Figure 3:**
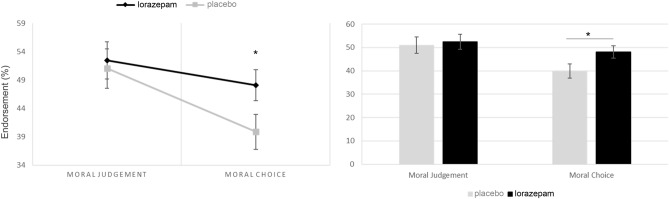
Dilemma judgements (i.e. % utilitarian responses) by treatment administration for the first-person choice and third-person judgement (*F*_1, 30_ = 4.98, *P* = 0.033).

In this analysis we controlled for harming outcomes across different moral perspective-taking conditions. In order to examine whether the lorazepam administration interact with different moral perspective-takings regardless of harming outcomes, we compared harm endorsement rates across various dilemma scenarios under both placebo and lorazepam conditions. This comparison specifically focused on scenarios viewed from the '1st person' and '3rd person' perspectives. Lorazepam administration significantly increased the 1st-person choice for the endorsement of harm (Lorazepam: 48.08 ± 3.05, Placebo: 39.86 ± 2.71, Z = 2.156, *P* = 0.031) but did not change the 3rd-person judgement (Lorazepam: 52.47 ± 3.45, Placebo: 51.02 ± 3.25, Z = 0.47, *P* = 0.638) (Table 2, Fig. 3). The Hypotheses 3 was supported.

### Summary

The analyses showed that the effect of lorazepam administration on moral endorsement of harm was modulated by the source of bringing about harm (personal vs. impersonal), as well as the moral perspective-taking (1st-person choice vs. 3rd-person judgement), but not by the inevitability of bringing about harm (evitable harm vs. inevitable harm).

### Informed consent

A written informed consent was obtained from all the participants, as well as were given a monetary compensation at the end of the study.

## General discussion

The objective of the present double-blind, crossover design, placebo-controlled study, was that of assessing the potential modulation of fear conditioning and aversive interoceptive processing on moral decision-making. For this purpose, pharmacological interventions using the GABAergic agonist lorazepam where employed in order to evaluate whether the drug’s anxiolytic effect exerted any influence over moral-decision making in general, or if this modulation was dependent on the inevitability of harm, or on perspective-taking. Our initial results showed that acute lorazepam administration increased the endorsement of harming actions for personal moral dilemmas, but not for impersonal moral dilemmas; thus, confirming our first hypothesis (H1), and replicating Perkins, et al.^11^’s findings. Nevertheless, our second hypothesis (2) was rejected, as we found that lorazepam administration had no significant effect on the endorsement of harm dependent of inevitability of harm. Last but not least, our results do corroborate our third hypothesis (H3), which states that when the moral dilemma task is modified to include perspective-taking (1st versus 3rd person perspective), lorazepam administration significantly increased the endorsement of harm in the 1st person choice-of-action probe, but not in the 3rd person moral judgement condition.

In line with Perkins, et al.^11^’s research, our findings demonstrate that lorazepam administration increases the endorsement of harming behaviors in personal moral dilemmas. In general, personal moral dilemmas tend to elicit a greater emotional arousal compared to impersonal moral dilemmas, as they highlight the individual’s direct bodily involvement when causing harm (e.g. having to physically push the man into the tracks in the footbridge dilemma, rather than just activating a switch in the trolley dilemma). Subsequently, this makes it more likely for the majority of participants to reject the endorsement of harmful behaviors proposed in the personal moral dilemmas^8,11,37,39,42–44^. However, we cannot conclude, as Perkins et al., that this is due to lorazepam administration incurring in an increment in ruthlessness. The moral dilemma task Perkins et al. utilized, and which was also used in the present research, is based on a particular type of dilemma, were participants are asked to decide whether they would sacrifice the life of a stranger in order to save the lives of numerous others; namely, ‘sacrificial dilemmas’. As such, and as other research has already argued^45^, sacrificial dilemmas tend to disregard the prosocial and altruistic aspects–e.g. impartial concern and fairness in regards to the welfare of everyone^46^– at the center of the utilitarian choices in these type of dilemmas. It is important to note though, that Perkins et al. do point out that ruthless decision-making is context-dependent: sometimes decisions that are considered ruthless benefit the greater good even when they are seen as objectively wrong (utilitarian perspective); and vice versa, sometimes ruthless decisions can be considered as objectively good when they’re in fact acting against the well-being of many (Kantian/deontological perspective) –e.g. telling the truth regarding the location of innocent victims of persecution, as lying is considered to be objectively wrong.

The sole fact that the anxiolytic drug lorazepam was capable of increasing the endorsement of harming behaviors in personal moral dilemmas, adds to the mounting evidence observing that anxiogenic states –or at least negative emotionality– have an important part to play in moral decision-making. Furthermore, and although past studies concerning emotion regulation have already shown that the downregulation or suppression of negative affects (such as fear) have the ability to foster risky and utilitarian moral preferences, and that, conversely, the upregulation of negative emotionality inhibits utilitarian moral choices and incurs in important risk-avoidance biases^2,47^; other research has observed that said risk-avoidant behaviors may be overly specific to anxiety, and not to negative affect as a whole^7^.

Moreover, the same mechanisms leading towards uncertainty and risky behaviors (i.e. actions made with no knowledge of their final consequences^48^) seem to underlie utilitarian moral choices. There is an important distinction to be made between anticipatory and anticipated emotions during the decision-making process^49^. Anticipatory emotions are those with immediate emotional, deep-rooted visceral reactions (e.g. anxiety, fear, dread) towards risk and uncertainty. Anticipated emotions are those expected to be experienced in the future as a consequence of an action; notwithstanding, these are shaped and informed by those experienced in the anticipatory state. While both deontological and utilitarian options arouse negative anticipatory emotions, when it comes to deontological choices, individuals know what to expect and what will happen exactly due to their actions (e.g. the trolley will kill the five workmen because they didn’t push the stranger to the tracks in the footbridge dilemma). On the contrary, for the utilitarian choice, the anticipated emotions and psychological burden are not clearly delineated (e.g. will they feel guilty? Will they regret their choice? How intense will these feelings be? For how long will they feel them?), hence, the utilitarian option is equated to a risky and anxiety-inducing choice^50^. If this is the case, there’s no surprise in lorazepam promoting utilitarian decision-making, not only in general, but also during personal and first-person perspective, as it would mediate both anticipatory and anticipated emotions. This is not only in line with the somatic marker hypothesis, but also with the risk-as-feelings hypothesis, with the caveat that contrary to the somatic marker hypothesis –which assumes that affect typically informs and complements the decision-making process–, the risk-as-feelings hypothesis posits that emotions may also dominate the decision-making process and generate behaviors which may deviate from what could be objectively seen as the “best course of action”^49^. In this sense, lorazepam would hamper this negative side of emotion and anxiety, correcting their course towards its informational aspect, and echoing the words of Luu, Tucker, and Derryberry^51^ who stated that “appropriate levels of anxiety reflect the highest level of normal motivational control of working memory, through which the operations of memory in planning and behavioral sequencing are continually linked with adaptive significance”^49^.

When it comes to the role of genetics and neurophysiology, a study using genetic data^52^ found that people with the short allele relative to those with the long allele of the 5-HTTLPR serotonin transporter polymorphism, tend to endorse more deontological moral choices than utilitarian ones. The short-allele of the 5-HTTLPR polymorphism transports less serotonin from the synaptic cleft back to the pre-synaptic neuron, thus, leaving more of said serotonin to interact with the serotonergic receptors; as such, the population of individuals with the short allele of this polymorphism tend to be more vulnerable towards developing neuroticism, negative emotionality, and finally, anxiety^53^. This is in line with studies showing that SSRIs are capable of increasing harm aversion and promoting prosocial behaviors^24^, as serotonin is a key component in processes transducing aversive cues into behavioral inhibition^25,26^. Moreover, envisioning harmful behaviors directed towards others engages neural areas such as the ventromedial prefrontal cortex, anterior cingulate cortex, the striatum and the amygdala, all of which possess dense serotonergic projections^27^. Although, and as its name states, the 5-HTTLPR is involved in the serotonergic system, whereas lorazepam is a GABAergic agonist, previous studies have found that both the serotonin transporter, as well as the GABAergic receptor Pro385Ser, are associated with neuroticism^54^. The short and long variants of the 5-HTTLPR predominantly affect the activity of the amygdala^53,55^, a region heavy in serotonergic activity where lorazepam has been observed to produce a significant inhibitory response^27,30,56–58^. Nonetheless, the interactions between the GABAergic and serotonergic systems are complex, and no conclusive results, to our knowledge, have elucidated any clear or exact relationship between these systems and their definitive implication in the negative emotionality related to anxiety^59,60^.

Interestingly, previous neuroscientific research using mice models^61^ has shown that noradrenergic reuptake inhibitors reduce behavioral reactivity to stress and modify central GABAergic neurotransmission in the hippocampus, the lateral septum and the amygdala, which indicate that central GABAergic pathways may modulate the effects of noradrenergic reuptake inhibitors on stress reduction. Nevertheless, these results seem at odds with the mechanisms proposed in some human studies, by which the noradrenergic beta-adrenoceptor antagonist propranolol would have an influence in moral decision-making through a physiological pathway leading to a reduction in emotional arousal^20,21^. Consequently, it is no surprise that other studies have contested such findings, casting doubt on the anxiolytic and stress-reducing effects of the beta-noradrenergic antagonist^22,62^. Notwithstanding, it is important to note that this can be due to a lack of inclusion of somatic marker data, as well as endophenotypes^53^, in the experimental design. It has been suggested that the anterior insula and the amygdala are implicated in anxiety due to their crucial role in interoception. Moreover, individuals with a higher susceptibility towards anxiety are more prone to perceive a heightened interoceptive prediction signal, which might originate as a consequence of a heightened signaling of salience elicited by the amygdala^31^.

A competing explanation to all the mentioned above, might be that benzodiazepines seem to decrease empathic responses and promote antisocial behavior^63^. This is in accordance with previous research showing that SSRI anxiolytics and antidepressants tend to inhibit empathy and induce emotional blunting^64–66^. A longitudinal fMRI study found that antidepressant interventions decreased neural responses in the bilateral anterior insular cortex and the anterior midcingulate cortex (two brain regions involved in the empathic response to pain), to the extent that previous findings attributing changes in empathy and emotional regulation to major depressive disorders might be actually associated with antidepressant treatment^67^. Furthermore, Benzodiazepines in general have been observed to increase aggressive behaviors in rats^68,69^. Flunitrazepam in particular, has been previously implicated in criminal and violent behaviors in male juvenile offenders, as it appears to suppress fear, enhance feelings of security and power, and increase aggression^63,70–72^. Nonetheless, all of the aforementioned studies with juvenile offenders assessed their outcomes in populations with a history of benzodiazepine addiction and abuse, while the present study was conducted using neurotypical and healthy volunteers. Additionally, said studies on juvenile offenders are observational in nature, as such a causal relationship between benzodiazepines, aggression and criminal behavior cannot be clearly established^63^.

The finding that endorsement of harm towards others diminishes with 1st person perspective-taking, and that lorazepam is capable of reverting such phenomenon, just shows that the old quarrel between Kantian/deontological and utilitarian moral reasoning might not be viable when the individual is physically involved in the predicament (being it in the real, actual space or the virtual, mental space) during moral decision-making. This is more evident with the finding that lorazepam does not have any significant effect on the endorsement of evitable or inevitable harm. Similar with the first hypothesis, a probable reason for this finding is that first person perspective, as well as moral personal dilemmas, increase negative emotionality, with the personal dilemmas enhancing it in an even greater manner than what was previously thought. Consequently, it doesn’t matter whether the harm is inevitable or evitable, lorazepam by itself might not be strong enough to override the excessive emotional arousal. Personal-inevitable moral dilemmas refer to moral dilemmas were an action involving direct physical contact, and which ends in harm or death for another person, is required in order to solve the predicament. As the name suggests, said harm or death is inevitable, independently of the responder’s negative or positive answer. One example is that of the “Rescue 911 dilemma”, where the leading character (being it the participants themselves in the 1st person perspective condition, or another person in the 3rd person perspective condition) is aboard a helicopter alongside other people (among them a patient), and which suddenly experiences a technical error. The only way of saving the crew on board is by lightening the load and throwing off the patient. If the main character decides not to throw off the patient, all people in the helicopter die. Conversely, if the main character throws the patient out, all the other people will be saved. Therefore, independently on whether the main character decides to throw out the patient or not, the patient still dies; hence, being the death or harm inevitable^44^. Nevertheless, it is important to note, that although not statistically significant, participants did show a stronger endorsement towards inevitable harm when compared to evitable harm, which is the most common outcome due to the principle of lesser evil^40,41^.

In line with the first hypothesis, an alternate explanation to the finding that the endorsement of harm decreased in the 1st person perspective-taking condition, but that lorazepam was capable of reverting said occurrence, might also be due to the aforementioned effects of benzodiazepines as empathic function inhibitors and emotional blunting agents^63^.

Some limitations of this study should be acknowledged. First, self-reported measures such as the moral dilemma task are very much able to be affected by individual differences regarding the willingness to please the experimenter, or to avoid the negative social reputation incurred by endorsing harming or unjust behaviors, as well as may also be affected by cognitive load and/or the participant’s ability to successfully imagine actions with various moral consequences^73^. As such, post-session questionnaires to assess the perceived interpersonal behavior of the experimenter might be helpful to evaluate the effects of response expectancy to the intervention in drug-placebo studies^74^. Second, this study makes no use of endophenotypes (e.g. EEG, fMRI, etc.); thus, this study is unable to elucidate the exact neural mechanisms by which lorazepam has its effect on the moral dilemma task and its different conditions. Nevertheless, this study makes use of pharmacological interventions with the GABA agonist lorazepam, whose anxiolytic effects and neuromodulation on brain regions such as the insula and the amygdala have already been well studied and documented^30,55,75^. Third, the study used solely sacrificial dilemmas to examine the effects of lorazepam on moral decision-making. Previous research has observed that sacrificial dilemmas are incapable of evaluating equality-based morality, as well as simultaneously assessing minimization of harm and maximization of benefits. Therefore, further research using different types of paradigms (e.g. minimal group, resource allocation, etc.) is highly warranted. Additionally, the latter types of paradigms are better suited to reflect real-life scenarios regarding moral decision-making^45^.

All in all, our findings were not only able to support those of Perkins et al.^11^, but also to complement and expand them. Our results suggest that the anxiolytic GABA agonist lorazepam is capable of downregulating key brain centers involved in fear conditioning and aversive interoceptive processing –i.e. the insula and amygdala. Consequently, modulating anticipatory and anticipated emotions, and reverting the negative emotionality generated by the physical involvement promoted during personal moral dilemmas, and moral dilemma tasks utilizing first-person perspective-taking.

In conclusion, this study highlights the role of GABAergic neuromodulation and negative affect, as well as the involvement of the amygdala and the insula, in moral decision-making processes. These findings are in agreement with research demonstrating that reason and emotion are not the only two components weighing in during moral decision-making, but rather there is a dynamic interplay between emotional salient information and first-hand physical, bodily and interoceptive inputs, all of which give rise to moral reasoning^3^. Furthermore, it is possible to see how applications of the dual-process model of moral decision-making –which regards moral reasoning as the product of two opposed subsystems competing against each other: one automatic and emotion-based against a rational and conscious-controlled^8,37^– would be dependent on the degree of physical, bodily involvement of the agent in different kinds of moral dilemma scenarios.

### Supplementary Information

Supplementary Information.

## Data Availability

All data generated or analyzed during this study are included in this published article and its supplementary information files. Further enquiries can be directed to the corresponding author.
