# Supplementary material for: Negative emotionality downregulation affects moral choice but not moral judgement of harm: a pharmacological study
Source: Sci Rep. 2024 Jan 12;14:1200. doi: 10.1038/s41598-024-51345-8 (PMC10786834; doi:10.1038/s41598-024-51345-8)
Supplement: Supplementary file 1 — Supplementary Information. [file 41598_2024_51345_MOESM1_ESM.docx]

**Supplementary results using parametric analysis in study 1**

**H1: Associations between source of bringing about harm and the effect of lorazepam.** An interaction between the source of bringing about harm (personal vs. impersonal) and the lorazepam effect was found. Lorazepam administration increased the endorsement of harming for the personal dilemmas but not for impersonal dilemmas in previous literature, this study replicated these findings. A 2 (administration: placebo vs. lorazepam) x 3 (dilemma type: non-moral vs. impersonal vs. personal) repeated ANOVA on the endorsement of harm revealed a significant interaction between administration and dilemma type (*F*_2, 80_ = 3.52, *P* = .03, pη^2^ = .081) as well as a main effect of dilemma type (*F*_2, 80_ = 45.55, *P* < .01, pη^2^ = .532). Endorsement of harm rate was lowest for the personal dilemmas (mean ± se: 36.96 ± 2.08), followed by the impersonal dilemmas (56.1 ± 2.23) and non-moral dilemmas (59.5 ± 1.39). Endorsement of harm for personal dilemmas was less likely compared to endorsement of harm for impersonal dilemmas (T = -7.25, *P* < .001), or the endorsement of non-moral choices (T = -8.03, *P* < .001). Lorazepam administration significantly increased the endorsement of harm in personal dilemmas (lorazepam vs. placebo: 40.33 ± 2.32 vs. 33.59 ± 2.51, T = 2.74, *P* = .009) but did not exert its effect for non-moral (55.28 ± 5.37 vs. 63.69 ± 5.47, T = -0.8, *P* = .428) or impersonal dilemmas (48.29 ± 4.47 vs. 63.90 ± 4.95, T = -1.88, *P* = .07). Hypotheses 1 was supported.

**H2: Associations between inevitability of bringing about harm and the effect of lorazepam.** In order to further examine whether the outcomes regarding harm in personal dilemmas interact with lorazepam administration, a new ANOVA model was created to test the endorsement for personal harm. A 2 (administration: placebo vs. lorazepam) x 2 (harming outcome: evitable vs. inevitable) repeated ANOVA on the endorsement of harm revealed a significant main effect of administration (*F*_1, 40_ = 8.0, *P* = .007, pη^2^ = .167), with lorazepam administration having stronger endorsement for personal harming (43.49 ± 2.53 vs. 35.74 ± 2.83), as compared to placebo administration, as well as a main effect of harming outcome (*F*_1, 40_ = 78.98, *P* < .001, pη^2^ = .664) where inevitable harm had stronger endorsement (53.85 ± 3.61 vs. 25.38 ± 1.65) when compared to evitable harm. However, the interaction between administration and harming outcome was not significant (*F*_1, 40_ = 0.6, *P* = .444, pη^2^ = .015). The effect size of lorazepam administration did not change across the inevitability of bringing about harm. Hypotheses 2 was not supported.

**Supplementary results using parametric analysis in study 1**

**H3: Associations between moral perspective-taking and the effect of lorazepam for moral dilemmas.** In this analysis we controlled for harming outcomes across different moral perspective-taking conditions. In a 2 (Perspective-taking: 1st-person choice vs. 3rd-person judgement) x 2 (administration: placebo vs. lorazepam) x 3 (dilemma type: impersonal vs. personal-evitable vs. personal-inevitable) repeated ANOVA on the endorsement of harming, there was a significant interaction between perspective-taking and administration (*F*_1, 30_ = 4.98, *P* = .033, pη^2^ = .142) in addition to the main effect of perspective-taking (*F*_1, 30_ = 8.01, *P* = .008, pη^2^ = .211), as well as the main effect of dilemma type (*F*_2, 60_ = 47.04, *P* < .001, pη^2^ = .611). Both personal-inevitable harm (58.67 ± 3.85, *P_Bonferroni corrected_* < .05) and impersonal harm (56.01 ± 2.31, *P_Bonferroni corrected_* < .05) showed stronger endorsement, as compared to personal-evitable harm (28.9 ± 2.3), whereas there was no difference between personal-inevitable harm and impersonal harm (*P* > .5). While the endorsement of harm was generally lower for the 1st-person choice (43.97 ± 2.24), as compared to the 3rd-person judgement (51.75 ± 2.84), the effect size of lorazepam administration was found to be larger for the 1st-person choice. Post-hoc comparisons showed that lorazepam administration significantly increased the 1st-person choice for the endorsement of harm (lorazepam vs. placebo: 48.08 ± 3.05 vs. 39.86 ± 2.71, *P* = .031) but did not change the 3rd-person judgement (lorazepam vs. placebo: 52.47 ± 3.45 vs. 51.02 ± 3.25, *P* = .687). The Hypotheses 3 was supported.

**Supplementary Table s1.** Results from Parametric analysis of T-test for moral permissibility (Study 1, N = 41) by dilemma type and drug condition.

|  | Placebo |  | Lorazepam |  |  |
| --- | --- | --- | --- | --- | --- |
| Measurements (%) | Mean ± SE |  | Mean ± SE | T value | P value |
| Nonmoral | 63.69 ± 5.47 |  | 55.28 ± 5.37 | -0.801 | 0.428 |
| Moral-impersonal | 63.9 ± 4.95 |  | 48.29 ± 4.47 | -1.879 | 0.068 |
| Moral-personal (all) | 33.59 ± 2.51 |  | 40.33 ± 2.32 | 2.738 | 0.009 |
| Moral-personal-evitable | 22.59 ± 2.23 |  | 28.17 ± 1.94 | 2.176 | 0.036 |
| Moral-personal-inevitable | 48.9 ± 4.83 |  | 58.8 ± 3.84 | 2.02 | 0.05 |

**Supplementary Table s2.** Results from Parametric analysis of T-test for moral judgement and choice (Study 2, N = 31) by dilemma type and drug condition.

|  | Placebo |  | Lorazepam |  |  |
| --- | --- | --- | --- | --- | --- |
| Measurements (%) | Mean ± SE |  | Mean ± SE | T value | P value |
| ***Moral judgement (3^st^ person-perspective)*** | | | | | |
| Moral judgement (all) | 51.02 ± 3.25 |  | 52.47 ± 3.45 | 0.407 | 0.687 |
| Moral-impersonal | 55.48 ± 6.06 |  | 63.87 ± 6.04 | 0.794 | 0.434 |
| Moral-personal-evitable | 33.87 ± 3.41 |  | 30.65 ± 3.2 | -1.063 | 0.296 |
| Moral-personal-inevitable | 63.71 ± 5.54 |  | 62.9 ± 5.54 | -0.124 | 0.902 |
| ***Moral choice (1st person-perspective)*** | | | | | |
| Moral choice (all) | 39.86 ± 2.71 |  | 48.08 ± 3.05 | 2.266 | 0.031 |
| Moral-impersonal | 44.03 ± 5.73 |  | 60.65 ± 6.12 | 1.567 | 0.127 |
| Moral-personal-evitable | 23.12 ± 2.96 |  | 27.96 ± 2.83 | 1.393 | 0.174 |
| Moral-personal-inevitable | 52.42 ± 5.72 |  | 55.65 ± 5.65 | 0.494 | 0.625 |
